# Angiopoietin2-mediated caveolin1 phosphorylation regulating transcytosis of renal tubular epithelial cell contributes to the occurrence of albuminuria under high glucose exposure

**DOI:** 10.1186/s12967-022-03388-6

**Published:** 2022-04-25

**Authors:** Jing Liu, Junxia Yao, Yi Zhao, Jinxuan Su, Jiajia Ye, Yumei Wang

**Affiliations:** 1 Department of Nephrology, Union Hospital, Tongji Medical College, Huazhong University of Science and Technology, Wuhan, 430022 China; 2 Center for Stem Cell Research and Application, Institute of Hematology, Union Hospital, Tongji Medical College, Huazhong University of Science and Technology, Wuhan, 430022 China; 3 The People’s Hospital of China Three Gorges University, The First People’s Hospital of Yichang, Yichang, 44300 China

**Keywords:** Diabetic kidney disease, Renal tubular epithelial cells, Transcytosis, Angiopoietin2, Caveolin1

## Abstract

**Background:**

Microlbuminuria is the earliest clinical evidence of diabetic kidney disease (DKD) and contributes to the induction and/or progression of DKD. Previous studies have shown that increased expression of angiopoietin2 (ANGPT2) is correlated with an increase in albuminuria. However, the critical role of ANGPT2 in albuminuria development remains unclear. Some studies have shown the significance of transcytosis in the occurrence of albuminuria, but it is unknown whether it takes place in albumin recycling in renal tubular cells of patients with DKD. Furthermore, the potential mechanism of this association also remains unclear.

**Methods:**

In this study, human renal tubular epithelial cells (HK-2) were cultured with high glucose in a Transwell plate to establish a transcytosis model, while C57BL/6 mice were intraperitoneally injected with streptozotocin to establish a DKD model. The expression of ANGPT2 and caveolin1 (CAV1) phosphorylation was dectected through immunohistochemistry and western blot analysis.

**Results:**

Transcytosis of albumin in renal tubular epithelial cells was downregulated after high glucose exposure, and increased expression of ANGPT2 and CAV1 phosphorylation both in vivo and in vitro was observed. Inhibition of ANGPT2 and CAV1 independently promoted transcytosis. Furthermore, ANGPT2 downregulation inhibited CAV1 phosphorylation, whereas CAV1 phosphorylation had no effect on the expression of ANGPT2.

**Conclusions:**

ANGPT2 reduces albumin transcytosis across renal tubular epithelial cells under high glucose conditions by activating CAV1 phosphorylation, thus increasing albuminuria in DKD. These findings suggested that ANGPT2 and CAV1 may be promising therapeutic targets for albuminuria in DKD.

**Supplementary Information:**

The online version contains supplementary material available at 10.1186/s12967-022-03388-6.

## Background

Albuminuria is the most prominent feature of diabetic kidney disease (DKD). Accumulating evidence has indicated that reduced reabsorption ability of renal tubular cells significantly contributes to the occurrence of albuminuria [1]; however, its mechanism has not been fully elucidated.

Reabsorption via receptor-mediated endocytosis in the proximal tubule, is the only documented process for tubular protein clearance [2]. Endocytosis is the preliminary step of transcytosis, which involves selective transcellular delivery from one surface of a polarized cell to the other via vesicular or tubular membrane carriers [3]. Depending on the endocytic pathway, transcytosis can be categorized into two major types: receptor-mediated selective transcytosis mediated by clathrin, and caveolae-mediated non-selective adsorptive transcytosis, which relies on the charge of the molecule and plasma membrane leading to the interactions between them [4]. Transcytosis has been shown to be involved in variety of cancers [3], stroke [4], liver injury [5], and kidney diseases [6–8]. In kidney disease, receptor-mediated endocytosis is responsible for the protein reabsorption and degradation of filtered albumin in the proximal tubule [1]. The Endocytosis malfunction efficiently abolishes proximal tubular albumin uptake in nephrotic mice, resulting in an increase in urinary albumin excretion [7]. However, the function and mechanism of tubular transcytosis in DKD remains unclear.

Increased angiopoietin-2(ANGPT2) serum levels, an endothelial dysfunction and injury biomarker, has been demonstrated to be associated not only with endothelial dysfunction, but also with DKD. Increased serum concentrations of ANGPT2 are associated with DKD in patients with type 1 diabetes [9]. The expression of angiopoietin-like 2 (ANGPTL-2) in DKD rats was elevated, and ANGPTL-2 knockdown attenuates DKD [10]. In human microvascular endothelial cells, advanced glycation products and hyperglycemia increased ANGPT2 production [11]. The induced expression of ANGPT2 complicated endothelial cell inflammation [12]. More importantly, podocyte-specific overexpression of ANGPT2 aggravated albuminuria in mice [13]. These studies highlight the involvement of ANGPT2 in the progression of DKD and albuminuria; however, the role of ANGPT2 in tubular albumin reabsorption is unclear.

Caveolae (CAVs) are specific shuttles that are presents on the cell surface and form small, flask-shaped plasma membrane invaginations that are responsible for transcytosis. Caveolae biogenesis and function depend on the coat proteins, caveolins and support proteins cavins. Caveolin-1(CAV1), a member of the caveolin family, is a bulb-shaped, 50–100 nm protein component of caveolae that is expressed in various tissues and implicated in several diseases. Expression of caveolins in trabecular meshwork cells and their implication in the pathogenesis of glaucoma has previously been described [14]. Growing evidence suggests that CAV1 acts as a vital chaperone to facilitate cellular lipid trafficking, homeostasis, endocytosis, and exocytosis. It helps deliver chemokines, albumin, and low- and high-density lipoproteins [15]. CAV1 has been proven to engage in albumin transcytosis in podocyte [16] and glomerular endothelial cells [17, 18]. CAV1 has also been shown to participate in DKD progression [19]. The role of CAV1 in tubular transcytosis in DKD and its association with albuminuria still require further research.

In the present study, we sought to elucidate the role of ANGPT2 mediated CAV1 phosphorylation in the regulation of transcytosis in renal tubular cells exposed to high glucose (HG) concentrations. It was hypothesized that upregulation of ANGPT2 may promote CAV1 phosphorylation, further inhibit transcytosis of renal tubular cells, and thereby decrease tubular albumin reabsorption, resulting in albuminuria during HG exposure. This study would provide more evidence to show that regulation of CAV1, and ANGPT2 may improve outcomes in albuminuria in DKD.

## Materials and methods

### Animals use and procedures

Twenty 8-week-old, adult, male C57BL/6 mice weighing 20–25 g were provided by HFK (Bioscience co. Ltd, Beijing, China). All mice had free access to water and standard chow. The mice were randomly divided into two groups: diabetic mice and normal control mice. Both groups were fasted overnight, and the diabetic group were subsequently injected intraperitoneally with streptozotocin (STZ, 150 mg/kg, Boster Biological Technology, Wuhan) once, as previously described [12]. The control group was administered the same volume of citrate. Three and seven days after the STZ injection, only mice with stable blood glucose levels > 16.7 mmol/L were classified as diabetic mice for the experiment. The mice were euthanized after 12 weeks. This study received approval from the Institutional Animal Care and Use Committee at Tongji Medical College, Huazhong University of Science and Technology (2019S2645).

### Assessment of metabolic and physiological parameters

Body weight and blood glucose level were measured biweekly, and blood and urine were collected before euthanasia. The blood glucose level and blood samples were assessed as described previously [12]. Urinary protein, serum creatinine and urinary creatinine were identified using an automatic biochemical analyzer (ADVIA 2400, Siemens, Erlangen, Germany) as previously described [12]. The creatinine clearance rate was calculated and expressed as µL/min/g.

### Histopathological analysis of kidney

Kidney tissue was excised, cut, fixed with paraformaldehyde and embedded in paraffin. Thereafter, the kidney tissue blocks were sliced into 4 µm-thick sections. To evaluate the kidney tissue damage, the sections were stained with haematoxylin–eosin (HE) and periodic acid -Schiff (PAS).

### Immunohistochemical (IHC) staining

Formalin-fixed paraffin-embedded sections were deparaffinized and hydrated using slide warmers and alcohol. For antigen retrieval, the sections were incubated in EDTA at 120 °C for 5 min, and then with 3% H_2_O_2_ for 15 min at room temperature. Nonspecific binding was blocked using 5% normal goat serum for 30 min. The slides were incubated with an anti-phospho-Caveolin-1 (CST3251; CST, Danvers, MA, USA) and anti-angiopoietin2 (ab153934; Abcam, Cambridge, MA, USA) antibody overnight at 4 °C. The sections were then washed in phosphate-buffered saline and incubated with biotinylated goat anti-rabbit antibody (Beyotime, Jiangsu, China) for 20 min. After being stained with reacted with 3,3’-diaminobenzidine (DAB, EnVision Detection Kit), the sections were dehydrated with an alcohol gradient, sealed with neutral gum, and observed under a light microscope.

### Cell culture, treatment and transfection

The human renal tubular epithelial cells (HK-2) cell line was obtained from the American Type Culture Collection (Manassas, VA, USA). Cells were cultured at 37 °C and 5% CO_2_ in Minimum Essential Medium **(**MEM, Hyclone, Logan, UT, USA) supplemented with 10% bovine serum albumin (BSA, Bioscience, Shanghai, China) and 1% penicillin–streptomycin in an incubator. For high glucose treatment, cells were cultured in 30 mmol/L d-glucose included medium.

### RNA transfection (siRNA and shRNA)

HK-2 cells were seeded in 6-well plates, grown to 30–40% confluence, and the medium was replaced with OPTI-MEM (Gibco, Thermo Fisher Scientific, MA, USA). Subsequently, Caveolin-1 siRNA (Ribobio, Guangzhou, China) was transfected using Lipofectamine 2000 (Invitrogen, Carlsbad, CA, USA) diluted in OPTI-MEM and the cells were gently agitated for 4 h. Thereafter, OPTI-MEM was replaced with normal medium and incubated for another 48 h in the absence or presence of 5 mmol/L or 30 mmol/L glucose. Thereafter, cells were collected for further experiments. For shRNA-targeted ANGPT2 transfection, lentiviruses expressing shRNA targeting ANGPT2 (Genechem, Shanghai, China) and the corresponding control vector were transfected as specified by the manufacturer. After 48 h, the transduction efficiency of the transfected cells was visualized using a fluorescence microscope (Nikon, Tokyo, Japan).

### Albumin uptake

Cells were plated into 6-well chamber slides (Corning, Palo Alto, CA, USA), incubated in 5.6 mM d-glucose and 30 mM d-glucose medium and grown until reaching 90–100% confluence. Albumin was labeled with fluorescein isothiocyanate (FITC, Bioscience, Shanghai, China) (FITC-BSA) as described previously [20]. After serum starvation for 4 h, cells were incubated with 100 μg/ml of FITC-BSA for 3 h. HK-2 was further fixed with 4% paraformaldehyde and permeabilized with 0.5% Triton X-100/PBS for 20 min, stained with DAPI to reveal cell nuclei and then slides were peeled off and sealed under coverslips. Images were taken with fluorescence microscope (Olympus BX51, Tokyo, Japan). Images were also taken after the cells were incubated with 100 μg/ml of FITC-BSA for 3 h and lysed with 2% Triton/PBS for 2 h, to detect the fluorescence intensity of lysis through the Olympus fluorescence microscope.

### Monolayer integrity for transcytosis assay

HK-2 was seeded into a Transwell 24-well plate (Corning, USA) and incubated as their normal growth condition. Upon reaching 100% confluence, the trans-epithelial electrical resistance (TEER) was measured to determine the integrity of HK-2 cell monolayer. The epithelial voltohmmeter, EVOM2™ (WPI, New Haven, CT, USA), was used to measure Ω using sterilized electrodes. Each well was measured more than 9 times to calculate the mean value. TEER was calculated as follows:

$$  {\text{TEER(}}\Omega *{\text{cm}}^{{2}} ) = \, [\Omega \left( {{\text{cellinserts}}} \right) \, - \Omega \left( {{\text{cell-freeinserts}} } \right)] \, *{\text{ Transwell filter area}} $$

If TEER > 40Ω*cm^2^, the cells could be used for transcytosis assays.

### Albumin transcytosis assay

The determined HK-2 was re-suspended in 5.5 mmol/L or 30 mmol/L d-glucose, moved to another Transwell 24-well plate and incubated for another 12 h. Then, HK-2 was incubated with 100 µg/ml FITC-BSA in the upper chambers of the Transwell, while the medium in the lower chambers of the Transwell was mixed with 100 µg/ml BSA. The chambers were removed and the medium in the lower chambers of the Transwell were mixed homogeneously. A fluorescence spectrophotometer (Infinite F200PRO; Tecan, Männedorf, Switzerland) was used to determine the fluorescence signal in the upper and lower chambers of the Transwell. Excitation and emission wavelengths for FITC-BSA were 490 and 520 nm, respectively.

### Western blot analysis

Renal tissue and HK-2 cells were collected and the total protein was extracted using a Radio Immunoprecipitation Assay (RIPA) lysis buffer (Beyotime, Jiangsu, China). A total of 50 µg of protein was loaded onto an 8–12% polyacrylamide gel and separated via sodium dodecyl sulphate (SDS)-polyacrylamide gel electrophoresis (PAGE). Thereafter, the proteins were transferred to polyvinylidene difluoride membranes (Millipore, Bedford, MA, USA). The membranes were blocked in 5% non-fat dried skimmed milk (Beyotime) for 1 h at room temperature and incubated with primary antibodies overnight at 4 °C. Finally, the membranes were incubated in a blocking buffer with secondary antibody (1:2500; Eric Biotechnology, Wuhan, China) for 2 h before detection. Glyceraldehyde-3-phosphate dehydrogenase (GAPDH) was used as the internal control. The primary antibodies used were: Angiopoietin2 (ab153934, Abcam), Caveolin-1 (D46G3) XP® Rabbit mAb (CST3267, CST), Phospho- Caveolin-1 (Tyr14) (CST3251, CST), GAPDH(ab8245, Abcam), DNM2 Polyclonal Antibody (14605-1-AP, Proteintech, Chicago, IL, USA), Syntaxin4 Polyclonal Antibody (14988-1-AP, Proteintech), Tie2 Polyclonal Antibody (19157-1-AP, Proteintech), SRC Polyclonal Antibody (11097-1-AP, Proteintech). The densitometric analysis of each band was performed by ImageJv1.8.0 software (NIH, Bethesda, MD, USA).

### Statistical analysis

GraphPad Prism 8.0 software was adopted for statistical analysis. All data were expressed as mean ± SEM. A Student’s t-test was used for the comparison of two groups and one-way analysis of variance (ANOVA) was used for more than two groups. Statistical significance was set as P < 0.05.

## Results

### High glucose inhibited albumin transcytosis in HK-2

**Fig. 1:**
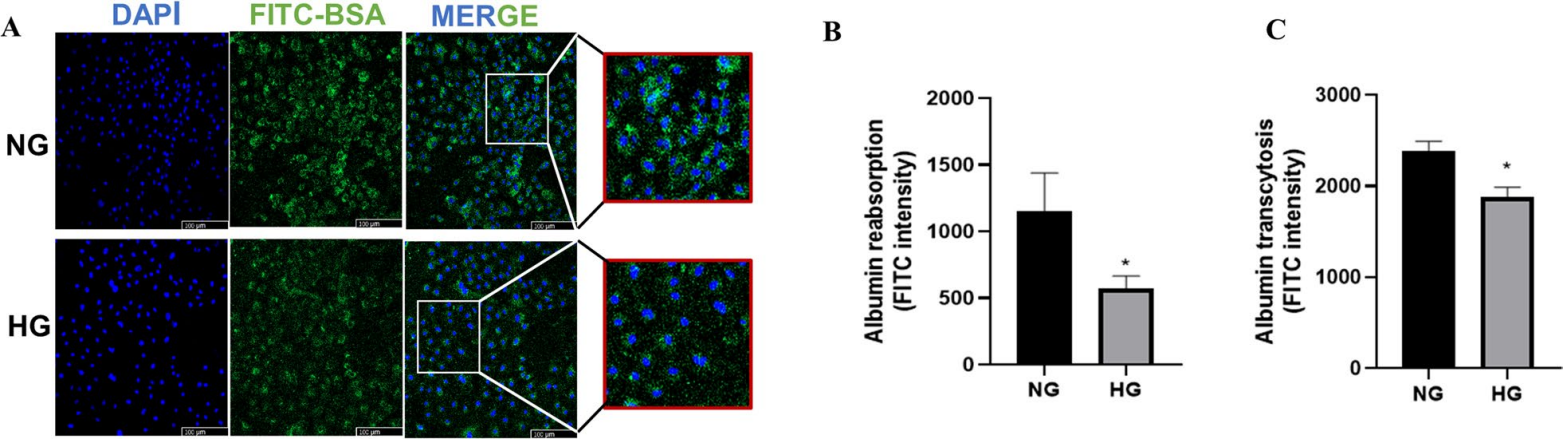
High glucose reduced albumin transcytosis in HK-2 cell. **A** Representative IF (immunoflurescence) images of albumin endocytosis. **B** FITC intensity in upper chamber of Transwell determined by Microplate System (n ≥ 3). ^*^P < 0.05 vs. NG (normal glucose). **C** FITC intensity in the lower chamber of the Transwell detected by Microplate System (n ≥ 3). ^*^P < 0.05 vs. NG

Although the mechanism of albuminuria in DKD is still elusive, albumin transcytosis has been reported to participate in albumin reabsorption and recycling in the kidney [1, 8]. To explore whether transcytosis occurs in renal tubular epithelial cells in DKD, we detected albumin endocytosis and transcytosis in HK-2 cells. Representative immunofluorescence images showed the endocytosis of intercellular FITC-BSA (Fig. 1A). While albumin endocytosis was high in normal glucose (NG) medium, endocytosis was significantly inhibited in cells cultured in HG medium. The FITC intensity in the HG-cultured lysate was much weaker than that in the NG lysate (Fig. 1B). Meanwhile, the FITC intensity in the lower chamber of the Transwell also decreased after HG administration (Fig. 1C). These results indicate that HG treatment inhibited endocytosis and transcytosis in HK-2 cells.

### ANGPT2 upregulation during HG exposure

**Fig. 2:**
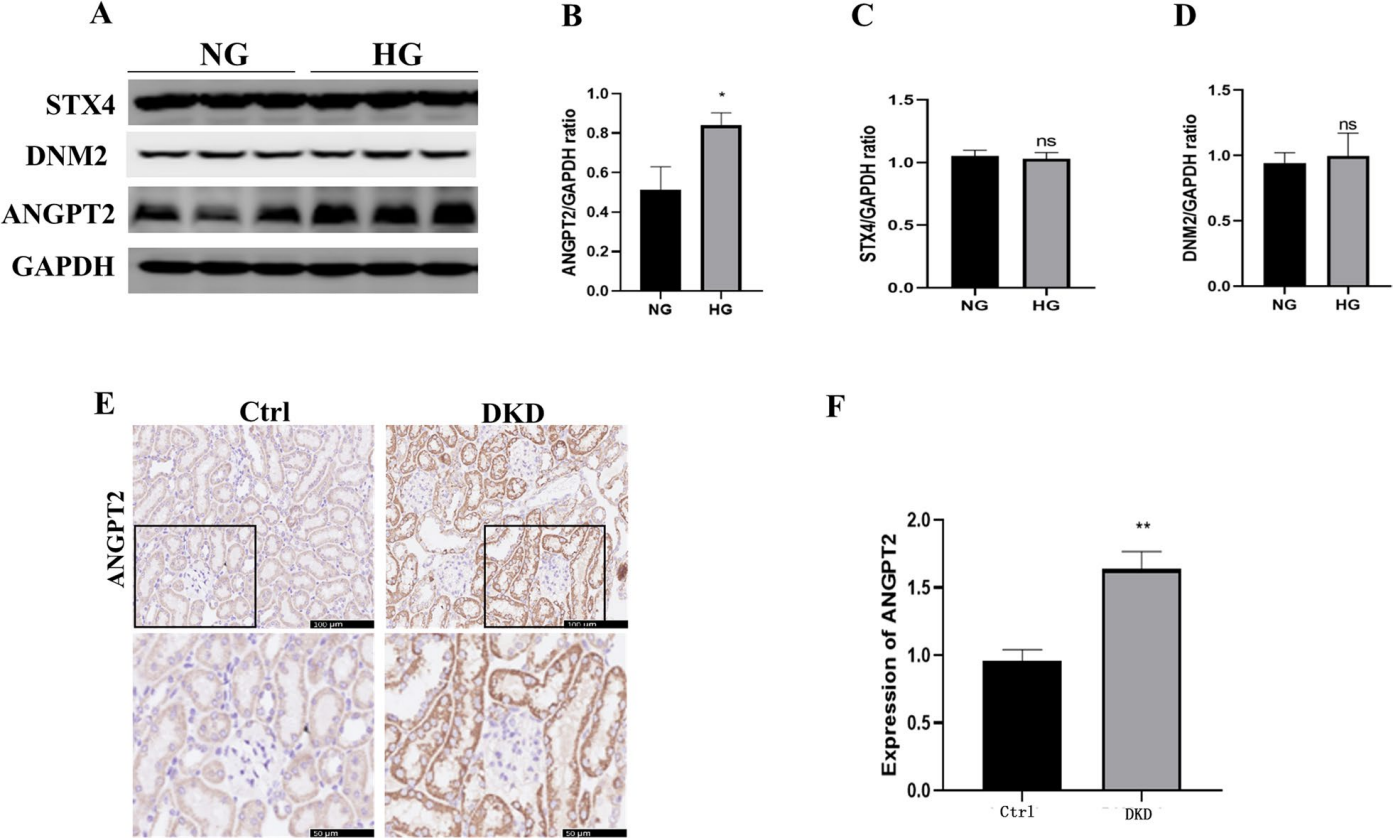
ANGPT2 upregulation during HG exposure. **A** Representative immunoblot of ANGPT2, STX4 and DNM2. **B** Densitometric analysis of ANGPT2 immunoblot signals (n ≥ 3). ^*^P < 0.05 vs. NG (normal glucose). **C** Densitometric analysis of STX4 immunoblot signals (n ≥ 3). No statistical significance vs. NG. **D** Densitometric analysis of DNM2 immunoblot signals (n ≥ 3). No statistical significance vs. NG. **E** Representative immunohistochemical (IHC) staining of ANGPT2 in diabetic kidney. **F** Statistical data of IHC staining of ANGPT2 (n ≥ 5). ^**^P < 0.01 vs. Control

ANGPT2 has been reported to be involved in diabetic nephropathy [12]. To investigate the potential relevance of ANGPT2 in DKD, the expression of ANGPT2 in HK-2 cells after HG incubation was detected using western blotting. Figure 2A shows the expression of ANGPT2 detected by western blotting, which indicated that ANGPT2 was upregulated in HK-2 cells of the HG group but not in the NG group (Fig. 2B). We also detected the expression of Syntaxin4 (STX4) [21] and dynamin-2 (DNM2) in HK-2 cells. However, the expression of STX4 (Fig. 2C) and DNM2 (Fig. 2D) was not significantly different between the groups. We further detected expression of ANGPT2 in the kidney tissue of mice with DKD. Compared with control mice, the blood glucose level (Additional file 1: Fig. S1A), ratio of the kidney weight/body weight (Additional file 1: Fig. S1B), serum creatinine (Additional file 1: Fig. S1C), and urine albumin/ creatinine ratio (Additional file 1: Fig. S1D) were all higher in diabetic mice. HE-staining revealed larger glomerular and mesangial volumes in study mice with nephropathy (Additional file 1: Fig. S1E). PAS staining revealed glomerular and tubular basement membrane thickening, mesangial matrix accumulation, and mesangial expansion (Additional file 1: Fig. S1F). These results are consistent with those of our previous reports [12] in which we exhibited the most representative characteristics of DKD. The ANGPT2 level was higher in the diabetic mice than in the control mice (Fig. 2E, F). Taken together, these findings indicate the probable involvement of ANGPT2 in HG-related kidney injury.

### CAV1 phosphorylation induced by HG administration

**Fig. 3:**
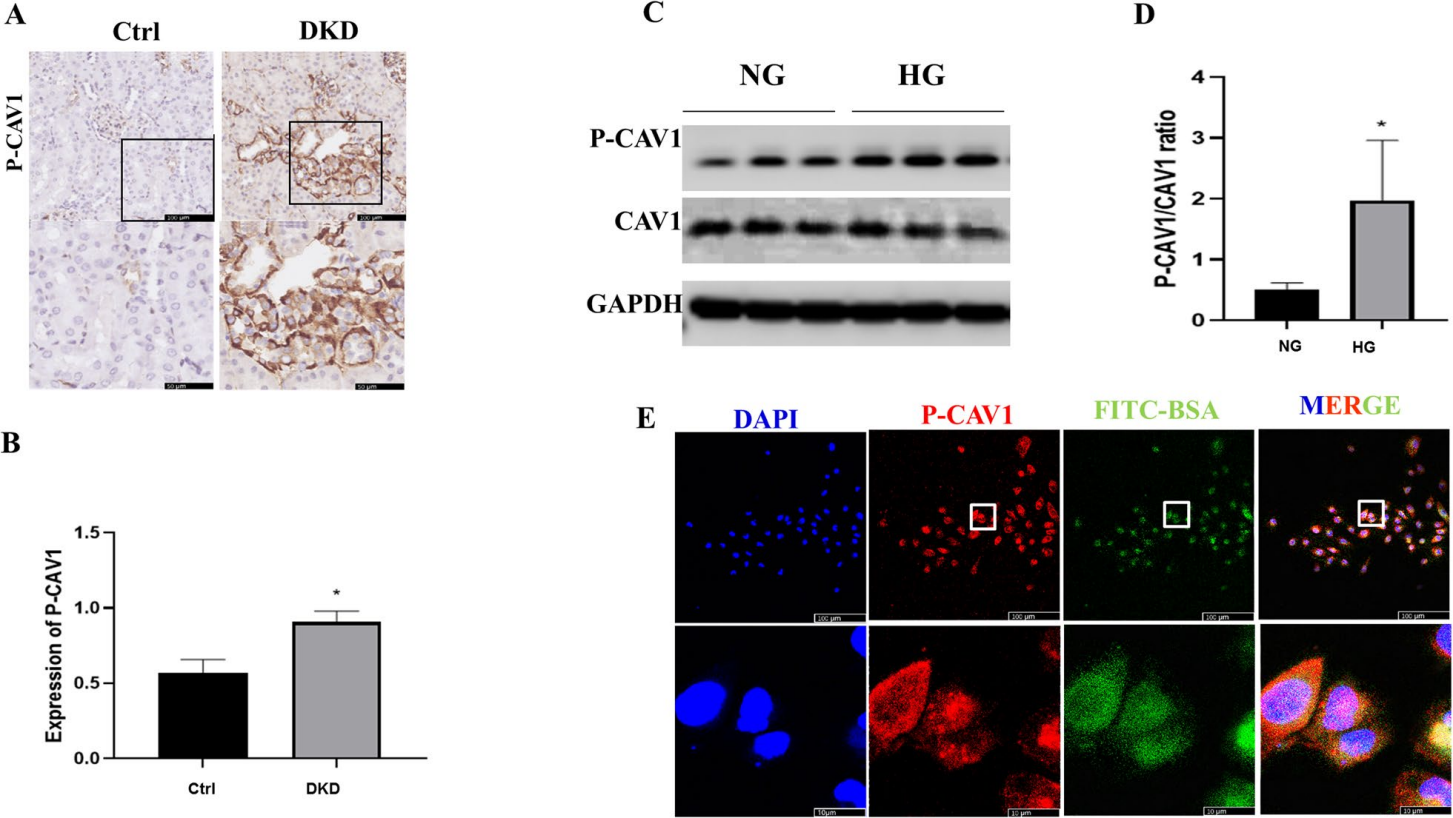
CAV1 phosphorylation was induced in HG-cultured HK-2 in vitro and diabetic kidney in vivo. **A** Representative immunohistochemical (IHC) staining images of P-CAV1. **B** Statistical data of IHC staining of P-CAV1 (n ≥ 5). ^**^ P < 0.01 vs. Control. **C** Representative immunoblot result of CAV1 and P-CAV1. **D** Densitometric analysis of phosphorylated-CAV1/ CAV1 ratio (n ≥ 3). ^*^P < 0.05 vs. NG. **E** Representative immunofluorescence (IF) images of CAV1 and absorbed FITC-BSA in HK-2 after cultured in high glucose (HG) medium

CAV1 has been documented to engage in transcytosis [22]; however, it has not previously been observed in diabetic tubular epithelial cells. We detected CAV1 expression in renal tubule cells following HG administration. CAV1 phosphorylation was upregulated in diabetic kidneys compared to that in control kidneys in study mice (Fig. 3A, B). Consistently, the expression of phosphor-CAV1 (P-CAV1) increased in HG-cultured HK-2 cells (Fig. 3C, D). Using immunofluorescence, we found that P-CAV1 and endocytosed albumin were co-localized in the cytoplasm of HK-2 cells (Fig. 3E), which implied that CAV1 was involved in albumin transcytosis.

### Inhibition of ANGPT2 promotes transcytosis

**Fig. 4:**
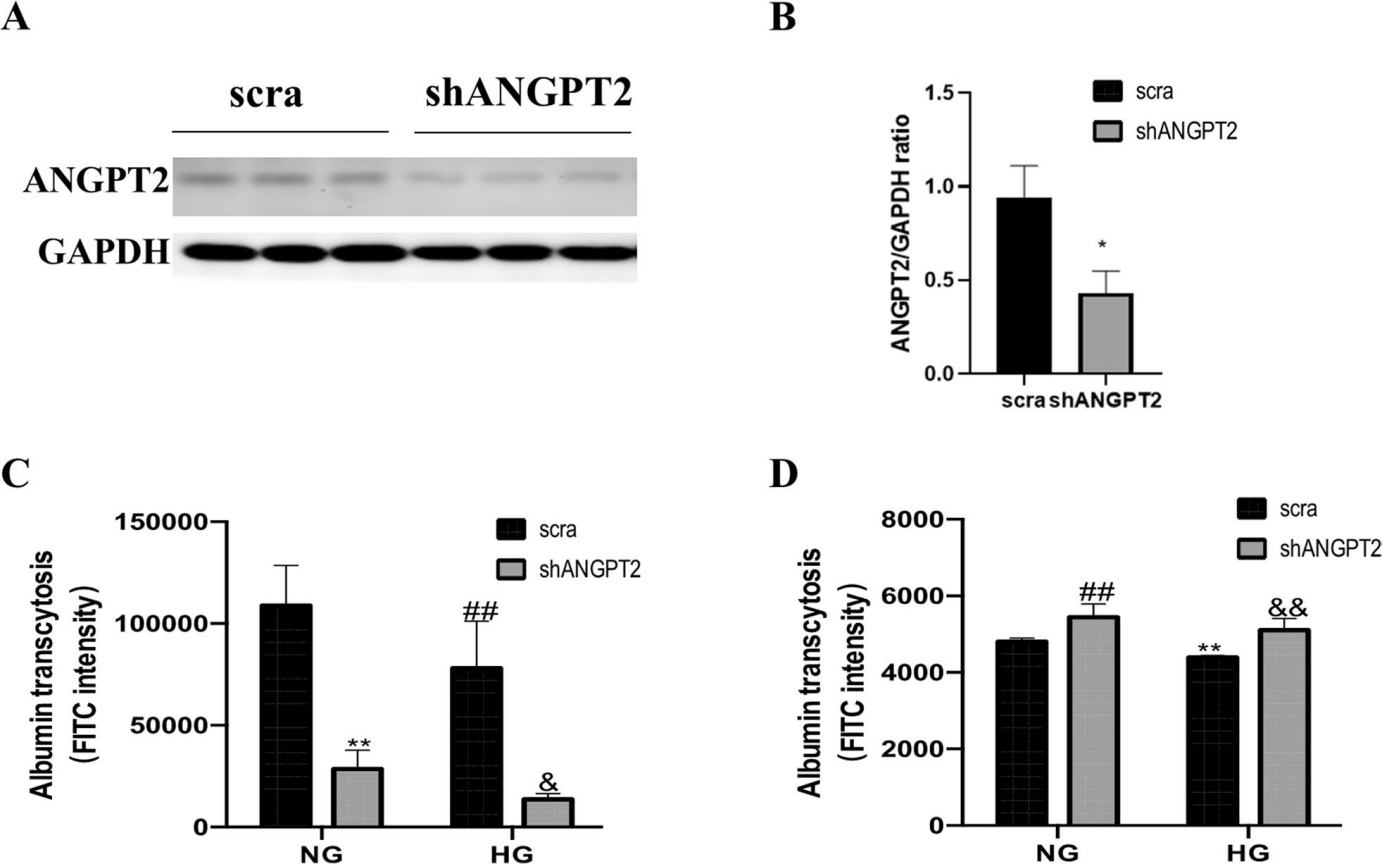
ANGPT2 downregulation promoted albumin transcytosis. HK-2 was transfected with shANGPT2, then the effect on albumin transcytosis was detected. **A** Representative immunoblot of ANGPT2 with shANGPT2 for 72 h. **B** Densitometric analysis of ANGPT2 immunoblot signals (n ≥ 3). ^*^P < 0.05 vs. scra (scramble shRNA). **C** Statistical IF signals in the upper chambers of Transwell (n ≥ 3). ^**^P < 0.01 vs. NG + scra, ^##^P < 0.01 vs. NG + scra, ^&^P < 0.05 vs. HG + scra. **D** Statistical immunofluorescence (IF) signals in in the lower chambers of Transwell (n ≥ 3). ^**^P < 0.01 vs. NG + scra, ^##^ P < 0.01 vs. NG + scra, ^&&^P < 0.01 vs. HG + scra

To investigate the role of ANGPT2 in transcytosis, we used a shRNA targeting ANGPT2. Western blot and quantitative analyses revealed decreased ANGPT2 expression after shANGPT2 transfection (Fig. 4A, B). We further examined the effect of shANGPT2 on albumin transcytosis using a Transwell system. FITC intensity in the upper chamber of the Transwell recede ANGPT2 even in NG conditions (Fig. 4C; shANGPT2 vs. scramble transfection). shANGPT2 further reduced FITC intensity in cells cultured in HG medium (Fig. 4C; shANGPT2 vs. scramble transfection). Meanwhile, the FITC intensity in the lower chamber of the Ttranswell was aggravated by shANGPT2 in NG conditions (Fig. 4D; shANGPT2 vs. scramble transfection). Once cultured in HG medium, shANGPT2 transfection increased albumin transcytosis, as detected by FITC intensity (Fig. 4D; shANGPT2 vs. scramble transfection). These results indicate that HG induced ANGPT2 expression, which may impede albumin transcytosis and led to albuminuria.

### Inhibition of CAV1 phosphorylation could facilitate transcytosis

**Fig. 5:**
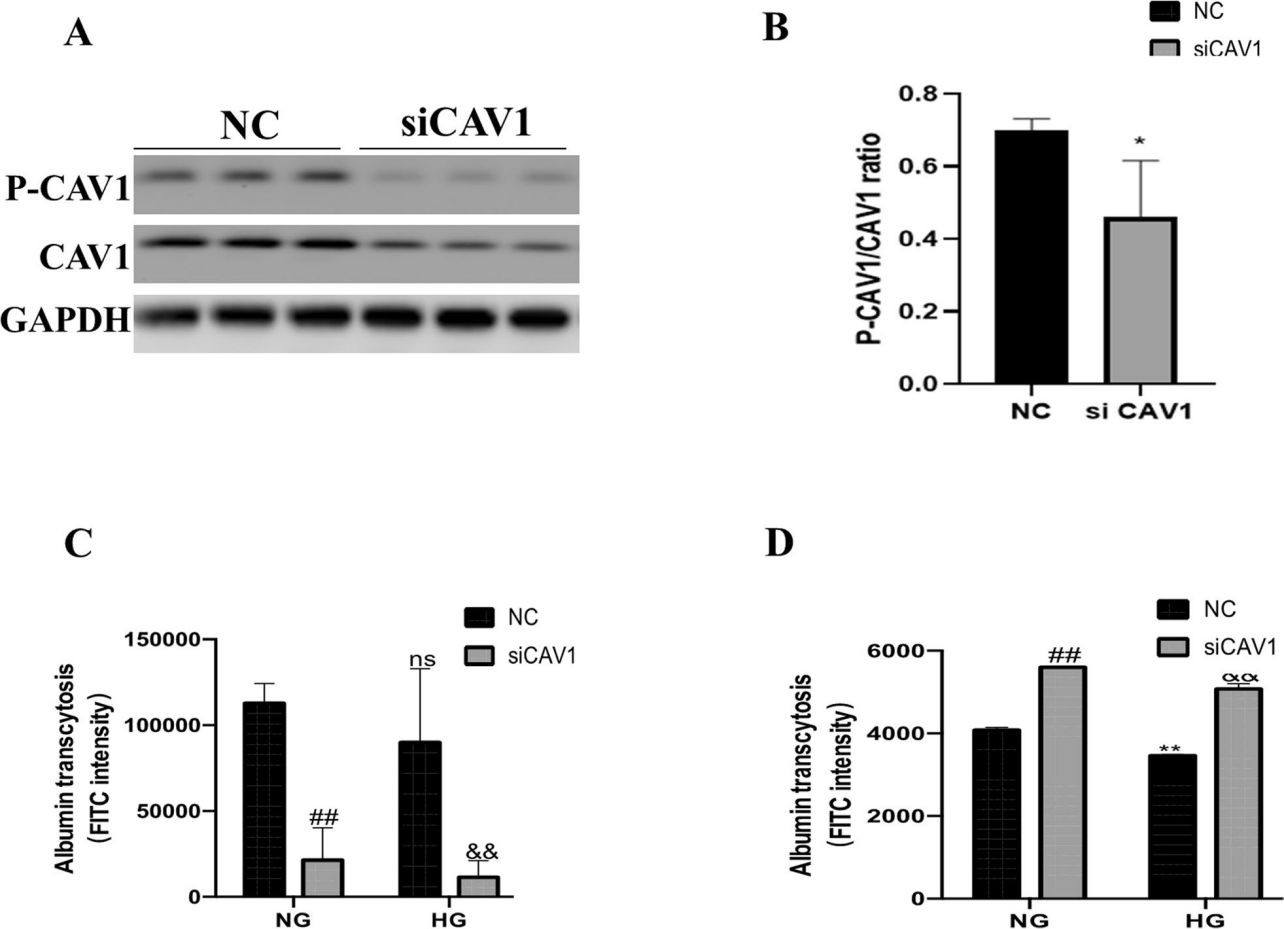
Inhibition of CAV1 facilitated albumin transcytosis in HK-2 cell. **A** Immunoblot of CAV1 and P-CAV1 in HK-2 after transfection with siCAV1 for 72 h. **B** Densitometric analysis of P-CAV1/CAV1 immunoblot signals (n ≥ 3). ^*^P < 0.05 vs. NC. **C** Statistical FITC intensity in the upper chamber of Transwell (n ≥ 3). ^##^P < 0.01 vs. NG + NC, ns vs. NG + NC, ^&&^P < 0.01 vs. HG + NC. **D** FITC intensity in the lower chamber of Transwell (n ≥ 3). ^##^P < 0.01 vs. NG + NC, ^**^P < 0.01 vs. NG + NC, ^&&^P < 0.01 vs. HG + NC. HG: high glucose; NC: normal control; NG: normal glucose

siRNA was used to inhibit the expression of CAV1. After siCAV1 transfection, the CAV1 and P-CAV1 were downregulated (Fig. 5A, B). Next, we validated the influence of siCAV1 on transcytosis using a Transwell chamber. The FITC intensity in the upper chamber of the Transwell was decreased by siCAV1 in NG (Fig. 5C; siCAV1 vs. NC). Once cultured in HG medium, FITC intensity was further reduced by siCAV1 transfection (Fig. 5C; siCAV1 vs. NC). The negative control (NC) in the upper chamber of the Transwell showed no obvious difference whether exposed to NG or HG (Fig. 5C). The FITC intensity in the lower chamber of the Transwell was increased by siCAV1 transfection in NG (Fig. 5D; siCAV1 vs. NC). After being cultured in HG medium, the FITC intensity after siCAV1 transfection was augmented in the lower chamber compared to the NC (Fig. 5D; HG siCAV1 vs. NC), which indicated the siCAV1 upregulated albumin transcytosis. Taken together, these data showed that CAV1 phosphorylation could block albumin transcytosis.

### ANGPT2 downregulation suppressed CAV1 phosphorylation

**Fig. 6:**
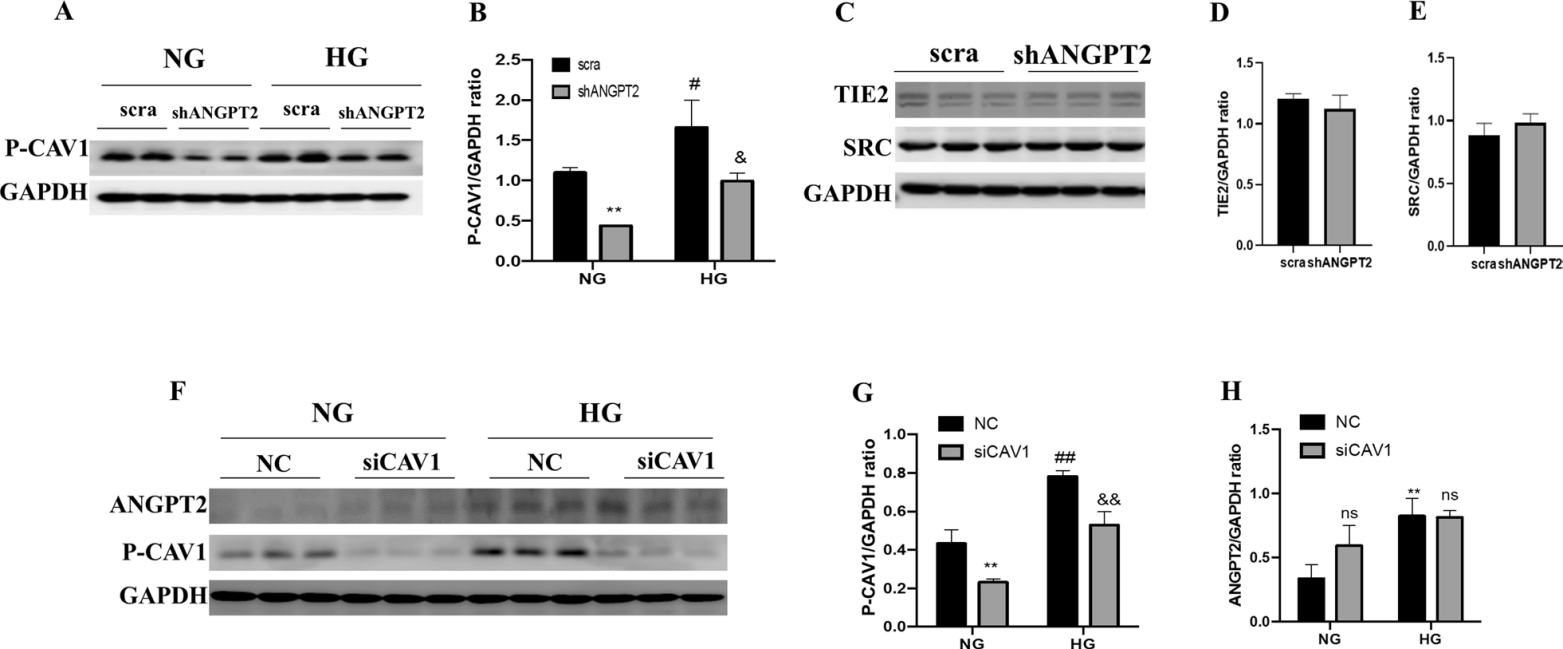
ANGPT2 downregulation suppressed CAV1 phosphorylation. **A** Immunoblot of P-CAV1 and CAV after transfection with shANGPT2 is shown in the figure. **B** Densitometric analysis ratio of P-CAV1/GAPDH (n ≥ 3). ^**^P < 0.01 vs. NG + scra, ^#^P < 0.05 vs. NG + scra, ^&^P < 0.05 vs. HG + scra. **C** HK-2 was transfected with shANGPT2, the immunoblot of TIE2 and Src. **D** Densitometric analysis of TIE2 and Src (n ≥ 3). No statistical significance was observed. **E** Densitometric analysis of Src (n ≥ 3). No statistical significance was observed. **F** Immunoblot of P-CAV1 and ANGPT2 after transfection with siCAV1. **G.** Densitometric analysis ratio of P-CAV1/GAPDH (n ≥ 3). ^**^P < 0.01 vs. NG + NC, ^##^P < 0.01 vs. NG + NC, ^&&^P < 0.01 vs. HG + NC. **H** Densitometric analysis of ANGPT2/GAPDH (n ≥ 3). ^**^P < 0.01 vs. NG + NC, ns vs. NG + NC, HG + NC. HG: high glucose; NC: normal control; NG: normal glucose

Given the involvement of both ANGPT2 and CAV1 in albumin transcytosis, we analysed their relationship in renal tubule cells. We used shANGPT2 to detect the role of in CAV1 and its phosphorylation. Upon shANGPT2 transfection, the expression of P-CAV1 was inhibited in NG or in HG (Fig. 6A). Statistical analysis of the P-CAV1/GAPDH ratio confirmed this result. In both the HG and NG medium, shANGPT2 transfection resulted in a reduction of P-CAV1/GAPDH (Fig. 6B). In addition, we detected the expression of TIE2 and SRC; however, the results showed no remarkable differences in their levels (Fig. 6C–E). We also determined the effect of siCAV1 transfection on ANGPT2. In both NG and HG media, siRNA transfection inhibited the expression of P-CAV1 (Fig. 6F), and the P-CAV1/GAPDH ratio decreased (Fig. 6G). However, siCAV1 transfection did not affect ANGPT2 expression in HK-2 cells in NG or HG media (Fig. 6H). In summary, shANGPT2 affected the expression of CAV1 phosphorylation, but siCAV1 showed no influence on ANGPT2, indicating that ANGPT2 may regulate albumin transcytosis by CAV1 phosphorylation.

## Discussion

Albuminuria is an independent risk factor for DKD. Induced glomerular injury [20, 23] and decreased albumin reabsorption due to impaired tubular could contribute to the generation of albuminuria. In the present study, we found that ANGPT2 reduced albumin transcytosis across renal tubular epithelial cells under HG conditions by activating CAV1 phosphorylation, thus increasing albuminuria.

Transcytosis is an important intracellular transport mechanism and is required for the delivery of albumin [22]. In the present study, we verified the activation of transcytosis for albumin in tubular cells and found that HG downregulated albumin transcytosis. Albumin transcytosis has been reported to take part in albumin reabsorption and recycling in the kidney [1, 8, 24]. Our findings indicate that a decrease in albumin transcytosis yields an increase in albuminuria, which is in accordance with the result that abnormal albumin transcytosis in tubular cells precedes the occurrence of proteinuric renal diseases [1].

Previous studies have delineated the importance of decreased albumin reabsorption in the presence of albuminuria [7, 13]. The Megalin-Cubilin complex mediates albumin endocytosis and reabsorption in the proximal tubules of DKD in mice [6], and megalin/cubilin knockout efficiently abolishes albumin uptake, resulting in an increase in urinary albumin excretion [7]. Neonatal Fc receptor (FcRn)-mediated albumin transcytosis has been described to function in a specific manner in albumin retrieval/reabsorption in tubular cells [13]. However, we did not find the specific albumin-binding protein involved in the transcytosis of albumin across renal tubular epithelial cells in the present study.

CAV1, the coat proteins of caveolae, is indispensable for caveolae formation [25], which has been proven to facilitate the profibrotic response to hyperglycemia in DKD [19] and participates in the transcytosis of glomerular endothelial cells [17, 18]. The results of recent investigations indicate that CAV-1 plays an important role in the association between diabetes and dementia [26]. It has also been documented to engage in endothelial cell transcytosis [22], but not in diabetic tubular epithelial cells. To date, there is still a lack of evidence regarding the correlation between albumin transcytosis and CAV1 expression in tubular cells.

Based on the involvement of transcytosis in albumin reabsorption, we investigated the role of CAV1 in regulating tubular cell transcytosis in DKD. Our results confirmed that CAV1 phosphorylation is upregulated in tubules of DKD mice and HG-cultured HK-2 cells, and the inhibition of CAV1 leads to the induction of albumin transcytosis. These results imply that CAV1 phosphorylation inhibits albumin transcytosis to aggravate albuminuria. Therefore, CAV1 could be a potential therapeutic target for the treatment of diabetic proteinuria.

In contrast to our results, in the endothelial cells of DKD, upregulated CAV1 phosphorylation enhanced albumin transcytosis [20]. The difference may be due to different cell types and flexible function of CAV1 phosphorylation. CAV1 phosphorylation affects the function and structure of caveolae and regulates transcytosis of albumin. It has been reported that the increase in CAV1 phosphorylation in rat tubular cells after Epithelial growth factor (EGF) administration significantly increased the number of caveolae [27]. Conversely, another study revealed that CAV1 phosphorylation is susceptible to degradation by the ubiquitin–proteasome pathway, which results in the instability of CAV1 [28], this further influences the activity of caveolae and transcytosis. Consistently, CAV1 phosphorylation results in the loss of 70S-CAV integrity, which makes it more accessible for degradation by ubiquitination enzymes, acyl-protein thioesterases and other degradative manner [29]. However, the roles and mechanisms of CAV1 phosphorylation in DKD require further investigation.

In addition, we identified the regulatory role of ANGPT2 in CAV1 mediated transcytosis which has been proven to be involved in albuminuria in our previous studies [12]. We found that shANGPT2 downregulates CAV1 phosphorylation and enhances albumin transcytosis. Since ANGPT2 is not a kinase, it cannot directly regulate CAV1 phosphorylation. ANGPT2 could stimulate CAV1 phosphorylation by depending on Src, the only kinase that has been proven to directly induce CAV1 Tyr 14 phosphorylation [30]. However, in this study, Src levels exhibited no difference between control and HG conditions. The ANGPT2 receptor and Tyrosine kinase receptors 2(TIE2)have been shown to participate in ANGPT2 regulation in different diseases [30–32]. ANGPT2 inhibit ANGPT-1 activation to block TIE2 phosphorylation in endothelial cells, which then inhibit angiogenesis [33]. Another study indicated that ANGPT2 can promote TIE2 phosphorylation [31]. However, our data showed no obvious difference of TIE2 between control and DKD groups. Therefore, the regulatory mechanism underlying ANGPT2/CAV1 phosphorylation requires further exploration.

**Fig. 7:**
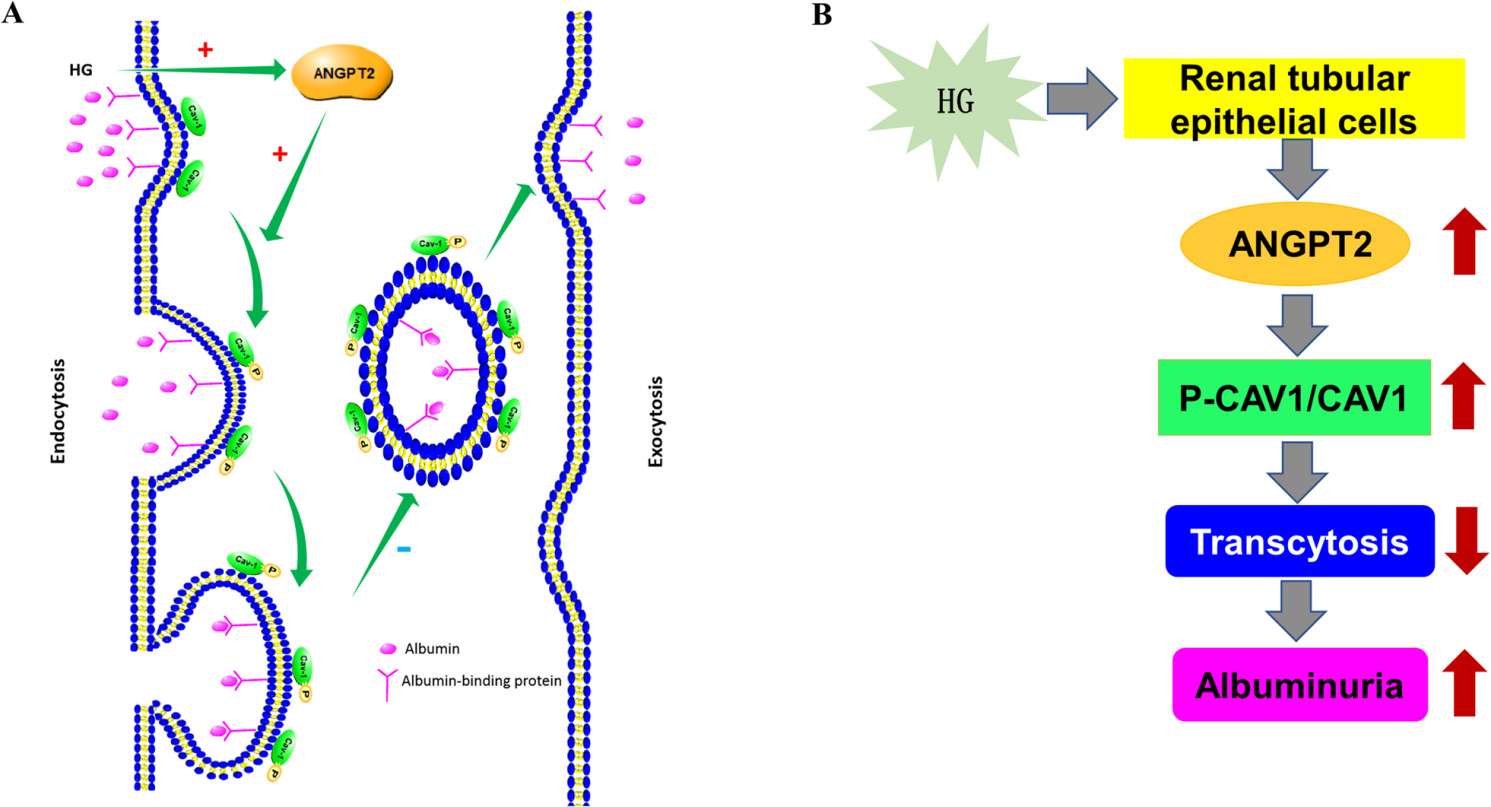
Schematic model for ANGPT2-mediated CAV1 phosphorylation in the inhibition of albumin transcytosis. Here we depict the model for albumin transcytosis under the regulation of ANGPT2 and CAV1. **A** ANGPT2/P- CAV1 signaling pathways involved in albumin transcytosis in HK-2 cells in HG. **B** Summary chart of this research findings. Following HG exposure, ANGPT2 is upregulated, which lead to increased CAV1 phosphorylation in tubular cells. CAV1 phosphorylation inhibited the albumin transcytosis. Angiopoietin2-mediated Caveolin1 phosphorylation blocked albumin transcytosis, which participates in the occurrence of diabetic kidney disease (DKD)-related albuminuria

In the current study, we found that albumin transcytosis was inhibited, whereas ANGPT2, CAV1 phosphorylation, and urine excretion of albumin were upregulated after HG exposure both in vitro and in vivo. ANGPT2 downregulation inhibited the CAV1 phosphorylation and increased albumin transcytosis (Fig. 7). Our findings suggest ANGPT2 activates CAV1 phosphorylation, which further blocks albumin transcytosis in tubular cells, leading to a decrease in albumin reabsorption and an increase in albumin excretion in urine. Strategies targeting ANGPT2 and CAV1 might be useful in treating DKD by blocking initial albuminuria.

It should be noted that this study has some limitations. We detected the regulation in vitro, but their function has not been fully explored in vivo in this study. Future studies will be directed at fully explicating the regulatory mechanisms, including the interrelation between ANGPT2 and CAV1 and CAV1 phosphorylation regulation on transcytosis. Meanwhile, more in vivo analyses need to be performed to detect the physiological roles and mechanism at global and tissue specific levels. These explorations will allow for further analysis of albuminuria in the progression of DKD. Interestingly, transcytosis has been explored to deliver drugs and genes for specific imaging and therapentics [3]. Given the vital role of transcytosis in kidney disease, it may be a promising therapeutic strategy for the treatment of kidney diseases.

## Conclusion

In summary, we found that ANGPT2 reduces albumin transcytosis across renal tubular epithelial cells under high glucose conditions by activating CAV1 phosphorylation, thus increasing albuminuria in DKD. Our study is the first to report that the role of ANGPT2-P-CAV1/CAV1 is correlated with albumin transcytosis inhibition in renal tubular cells in DKD. These findings suggest that ANGPT2 and CAV1 may be possible targets for albuminuria treatment in patients with DKD.

## Supplementary Information

**Additional file 1.** STZ injection induced diabetic kidney disease in C57BL7mice.** 1A** Blood glucose level (BG).** 1B** Ratio of the kidney weight/body weight (KBWR).** 1C** Serum creatinine (Scr).** 1D** Urine albumin/creatinine ratio (UACR).** 1E** Representative HE (Haematoxylin-eosin) staining images of kidney.** 1F** Representative PAS (periodic acid-Schiff) staining images of kidney. Data are expressed as mean±SD (n ≥ 5). *P<0.05 vs. control group (Ctrl), ** P<0.01 vs. Ctrl, *** P<0.001 vs. Ctrl.

## Data Availability

The datasets used and/or analysed during the current study are available from the corresponding author on reasonable request.

## Abbreviations

ANGPT2: Angiopoietin2
BSA: Bovine serum albumin
CAV1: Caveolin1
DKD: Diabetic kidney disease
DAB: Diaminobenzidine
DNM2: Dynamin-2
FITC: Fluorescein isothiocyanate
GAPDH: Glyceraldehyde-3-phosphate dehydrogenase
HE: Haematoxylin–eosin
HG: High glucose
NG: Normal glucose
HK-2: Human renal tubular epithelial cells
IHC: Immunohistochemical
MEM: Minimum Essential Medium
PAS: Periodic acid–Schiff
P-CAV1: Phosphor-CAV1
STX4: Syntaxin 4
TEER: Trans-epithelial electrical resistance
TIE2: Tyrosine kinase receptors 2

## References

[1] Dickson LE, Wagner MC, Sandoval RM (2014). The proximal tubule and albuminuria: really!. J Am Soc Nephrol.

[2] Amsellem S, Gburek J, Hamard G (2010). Cubilin is essential for albumin reabsorption in the renal proximal tubule. J Am Soc Nephrol.

[3] Liu X, Jiang J, Meng H (2019). Transcytosis—an effective targeting strategy that is complementary to "EPR effect" for pancreatic cancer nano drug delivery. Theranostics.

[4] Ayloo S, Gu C (2019). Transcytosis at the blood-brain barrier. Curr Opin Neurobiol.

[5] Pyzik M, Rath T, Kuo TT (2017). Hepatic FcRn regulates albumin homeostasis and susceptibility to liver injury. Proc Natl Acad Sci USA.

[6] Zhou L, Liu F, Huang XR (2011). Amelioration of albuminuria in ROCK1 knockout mice with streptozotocin-induced diabetic kidney disease. Am J Nephrol.

[7] Weyer K, Andersen PK, Schmidt K (2018). Abolishment of proximal tubule albumin endocytosis does not affect plasma albumin during nephrotic syndrome in mice. Kidney Int.

[8] Tenten V, Menzel S, Kunter U (2013). Albumin is recycled from the primary urine by tubular transcytosis. J Am Soc Nephrol.

[9] Sokolovska J, Stefanovics J, Gersone G (2020). Angiopoietin 2 and Neuropeptide Y are associated with diabetic kidney disease in type 1 diabetes mellitus. Exp Clin Endocrinol Diabetes.

[10] Yang S, Zhang J, Wang S (2017). Knockdown of angiopoietin-like protein 2 ameliorates diabetic nephropathy by inhibiting TLR4. Cell Physiol Biochem.

[11] Puddu A, Sanguineti R, Maggi D (2019). advanced glycation end-products and hyperglycemia increase angiopoietin-2 production by impairing angiopoietin-1-Tie-2 system. J Diabetes Res.

[12] Luo C, Li T, Zhang C (2014). Therapeutic effect of alprostadil in diabetic nephropathy: possible roles of angiopoietin-2 and IL-18. Cell Physiol Biochem.

[13] Davis B, Dei Cas A, Long DA (2007). Podocyte-specific expression of angiopoietin-2 causes proteinuria and apoptosis of glomerular endothelia. J Am Soc Nephrol.

[14] Surgucheva I, Surguchov A (2011). Expression of caveolin in trabecular meshwork cells and its possible implication in pathogenesis of primary open angle glaucoma. Mol Vis.

[15] Vykoukal J, Fahrmann JF, Gregg JR (2020). Caveolin-1-mediated sphingolipid oncometabolism underlies a metabolic vulnerability of prostate cancer. Nat Commun.

[16] Dobrinskikh E, Okamura K, Kopp JB (2014). Human podocytes perform polarized, caveolae-dependent albumin endocytosis. Am J Physiol Renal Physiol.

[17] Moriyama T, Takei T, Itabashi M (2015). Caveolae may enable albumin to enter human renal glomerular endothelial cells. J Cell Biochem.

[18] Moriyama T, Sasaki K, Karasawa K (2017). Intracellular transcytosis of albumin in glomerular endothelial cells after endocytosis through caveolae. J Cell Physiol.

[19] Van Krieken R, Krepinsky JC (2017). Caveolin-1 in the pathogenesis of diabetic nephropathy: potential therapeutic target?. Curr DiabRep.

[20] Wu D, Yang X, Zheng T (2016). A novel mechanism of action for salidroside to alleviate diabetic albuminuria: effects on albumin transcytosis across glomerular endothelial cells. Am J Physiol Endocrinol Metab.

[21] Evesson FJ, Peat RA, Lek A (2010). Reduced plasma membrane expression of dysferlin mutants is attributed to accelerated endocytosis via a syntaxin-4-associated pathway. J Biol Chem.

[22] Sowa G (2012). Caveolae, caveolins, cavins, and endothelial cell function: new insights. Front Physiol.

[23] Batchu SN, Majumder S, Bowskill BB (2016). Prostaglandin I2 receptor agonism preserves β-cell function and attenuates albuminuria through nephrin-dependent mechanisms. Diabetes.

[24] Molitoris BA (2014). Using 2-photon microscopy to understand albuminuria. Trans Am Clin Clim Assoc.

[25] Surguchov A (2020). Caveolin: a new link between diabetes and AD. Cell Mol Neurobiol.

[26] Chidlow JH, Sessa WC (2010). Caveolae, caveolins, and cavins: complex control of cellular signalling and inflammation. Cardiovasc Res.

[27] Orlichenko L, Huang B, Krueger E (2006). Epithelial growth factor-induced phosphorylation of caveolin 1 at tyrosine 14 stimulates caveolae formation in epithelial cells. J Biol Chem.

[28] Yoon HJ, Kim DH, Kim SJ (2019). Src-mediated phosphorylation, ubiquitination and degradation of caveolin-1 promotes breast cancer cell stemness. Cancer Lett.

[29] Busija AR, Patel HH, Insel PA (2017). Caveolins and cavins in the trafficking, maturation, and degradation of caveolae: implications for cell physiology. Am J Physiol Cell Physiol.

[30] Sun Y, Hu G, Zhang X (2009). Phosphorylation of caveolin-1 regulates oxidant-induced pulmonary vascular permeability via paracellular and transcellular pathways. Circ Res.

[31] Mirando AC, Shen J, Silva RLE (2019). A collagen IV-derived peptide disrupts α5β1 integrin and potentiates Ang2/Tie2 signaling. JCI insight.

[32] Kim M, Allen B, Korhonen EA (2016). Opposing actions of angiopoietin-2 on Tie2 signaling and FOXO1 activation. J Clin Investig.

[33] He MF, Liu L, Ge W (2009). Antiangiogenic activity of *Tripterygium wilfordii* and its terpenoids. J Ethnopharmacol.

