# Supplementary figures and images for: Angiopoietin2-mediated caveolin1 phosphorylation regulating transcytosis of renal tubular epithelial cell contributes to the occurrence of albuminuria under high glucose exposure
Source: J Transl Med. 2022 Apr 25;20:185. doi: 10.1186/s12967-022-03388-6 (PMC9036792; doi:10.1186/s12967-022-03388-6)

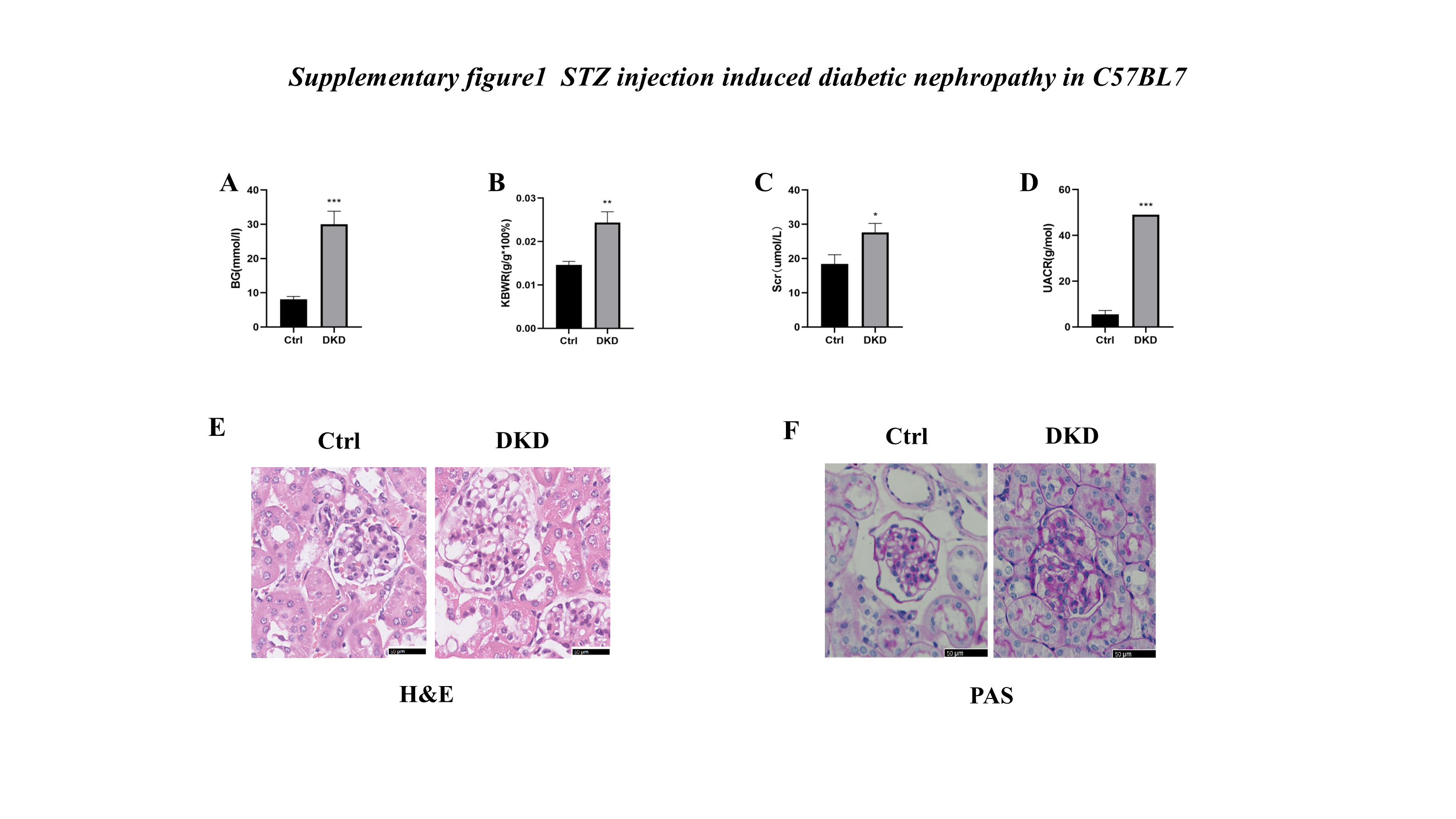

Supplement: Supplementary file 1 — Additional file 1. STZ injection induced diabetic kidney disease in C57BL7mice. 1A Blood glucose level (BG). 1B Ratio of the kidney weight/body weight (KBWR). 1C Serum creatinine (Scr). 1D Urine albumin/creatinine ratio (UACR). 1E Representative HE (Haematoxylin-eosin) staining images of kidney. 1F Representative PAS (periodic acid-Schiff) staining images of kidney. Data are expressed as mean±SD (n ≥ 5). *P<0.05 vs. control group (Ctrl), ** P<0.01 vs. Ctrl, *** P<0.001 vs. Ctrl. [file 12967_2022_3388_MOESM1_ESM.tif]
